# MR Pulse Sequences for Parathyroid Adenoma Imaging Using [^18^F]fluorocholine PET/MR in Primary Hyperparathyroidism

**DOI:** 10.1007/s13139-025-00961-x

**Published:** 2025-11-03

**Authors:** Shaza K. Isenschmid, Jan A. Schaab, Antonio G. Gennari, Junko Inoue Inukai, Grégoire B. Morand, Simon A. Mueller, Niels J. Rupp, Bert-Ram Sah, Victoria Schober, Urs J. Muehlematter, Petra Petranović Ovčariček, Luca Giovanella, Virginia Liberini, Philipp A. Kaufmann, Michael Messerli, Martin W. Huellner

**Affiliations:** 1 Department of Nuclear Medicine, University Hospital of Zurich, Rämistrasse 100, Zurich, 8091 Switzerland; 2 University of Zurich, Pestalozzistrasse 3, Zurich, 8032 Switzerland; 3 Department of Neuropediatrics, University Children’s Hospital of Zurich, Lenggstrasse 30, Zurich, 8008 Switzerland; 4 Department of Otorhinolaryngology, Head and Neck Surgery, University Hospital of Zurich, Frauenklinikstrasse 24, Zurich, 8091 Switzerland; 5 Department of Pathology and Molecular Pathology, University Hospital of Zurich, Schmelzbergstrasse 12, Zurich, 8091 Switzerland; 6 Department of Diagnostic, Interventional, and Pediatric Radiology, Inselspital, University of Bern, Rosenbühlgasse 27, Bern, 3010 Switzerland; 7 Department of Oncology and Nuclear Medicine, University Hospital Center Sestre Milosrdnice, Vinogradska cesta 29, Zagreb, 10000 Croatia; 8 School of Medicine, University of Zagreb, Šalata 3, Zagreb, 10000 Croatia; 9 Department of Nuclear Medicine, Gruppo Ospedaliero Moncucco SA, Clinica Moncucco, Via Soldino 5, Lugano, 6900 Switzerland; 10 Department of Nuclear Medicine, Azienda Ospedaliera S Croce e Carle Cuneo, Via Michele Coppino 26, Cuneo, Piemonte, 12100 Italy; 11 Department of Radiology, Kobe University Graduate School of Medicine, 7- 5-1, Kusunoki-cho, Chuo-ku, Kobe, 650-0017 Hyogo Japan

**Keywords:** Choline, Hyperparathyroidism, primary, Magnetic resonance imaging, Parathyroid glands, Positron-Emission tomography

## Abstract

**Purpose:**

Currently, no standardized anatomic magnetic resonance (MR) imaging protocol exists for detecting parathyroid adenomas. We analyzed various MR pulse sequences to evaluate their performance in visualizing histopathologically confirmed parathyroid adenomas in patients with primary hyperparathyroidism (pHPT) undergoing [^18^F]fluorocholine positron emission tomography (PET)/MR.

**Methods:**

This retrospective study included 128 adenomas in 110 patients with biochemically confirmed pHPT who underwent [^18^F]fluorocholine PET/MR at our institution between December 2020 and October 2023. Two radiologists independently characterized the lesions (as upper pole, lower pole, or ectopic adenomas). Surgical reports and histopathology served as reference standard. Lesion conspicuity, delineation, and size were compared on axial T1-weighted fast spin echo sequence (T1w FSE) and axial T2-weighted iterative decomposition of water and fat with echo asymmetry and least-squares estimation (IDEAL) sequence with water image reconstruction (T2w FSE flex water). Interreader agreement was determined using Cohen’s kappa; differences were analyzed using Wilcoxon signed-rank test.

**Results:**

Parathyroid adenomas had significantly higher conspicuity, superior delineation, and were larger (*p* < 0.001) on T2w FSE flex water images compared to T1w FSE images. While these differences were maintained in the subgroup analysis for upper and lower pole adenomas, ectopic adenomas were of similar size on both MR pulse sequences (*p* = 0.646).

**Conclusion:**

T2w FSE flex water offers significantly better visualization of parathyroid adenomas compared to T1w FSE, especially in orthotopic lesions. These results support the targeted use of such a limited MR protocol as part of PET/MR in the preoperative assessment of patients with pHPT.

## Introduction

Primary hyperparathyroidism (pHPT) is a common endocrine disorder, predominantly prevalent in the elderly. In individuals over the age of 70, the prevalence is approximately 1.5%, with an incidence reaching up to 196 cases per 100,000 population in the 70–79-year age group [1–6].

Pathophysiologically, pHPT is most commonly caused by the autonomous, benign overproduction of parathyroid hormone (PTH), typically due to the presence of one or more parathyroid adenomas. Less frequently, the condition arises from parathyroid hyperplasia – which is not reliably distinguishable from adenomas on histopathology – or, in rare cases, parathyroid carcinoma [2–7]. Clinically, this hormonal dysregulation leads to hypercalcemia, with complications including nephrolithiasis, renal impairment, osteoporosis, and neuropsychiatric symptoms [2–7]. At present, parathyroidectomy targeting the hyperfunctioning glands remains the only curative treatment [3].

Traditional first-line imaging modalities, such as ultrasound and ^99m^Tc-methoxy isobutyl isonitrile single-photon emission computed tomography (SPECT)/computed tomography (CT) [8–16], demonstrate reasonable sensitivity (approximately 80%). Still, their performance is often limited in patients with atypical gland locations, small adenomas, or multiglandular disease [17, 18]. 4D-CT has been reported to offer reasonable localization [11, 19–24]. The advent of [^18^F]fluorocholine positron emission tomography (PET)/CT and PET/magnetic resonance (MR) imaging has markedly improved parathyroid imaging, offering high sensitivity and lesion detectability, even in complex or inconclusive cases [25–31].

In recent years, [^18^F]fluorocholine PET/CT has become a first-line imaging modality (alongside ultrasound). PET/MR systems raise the possibility of further improving diagnostic accuracy by combining the high sensitivity of PET with the superior soft-tissue contrast of MR, at reduced radiation exposure [32]. Reported sensitivities for PET/MR range from 84% to 100% with consistently high positive predictive values, supporting its role as a “one-stop shop” for preoperative localization of hyperfunctioning parathyroid tissue [33–40].

However, PET/MR remains limited by a lack of standardized imaging protocols, especially concerning the optimal MR pulse sequences, which are crucial to differentiate parathyroid glands from adjacent cervical structures such as lymph nodes [41].

High soft tissue contrast is essential and best achieved using sequences such as T1-weighted Dixon, T2-weighted fat-suppressed (FS), and short tau inversion recovery (STIR) sequences. Several studies suggest that combining morphological MR sequences with [^18^F]fluorocholine PET allows for adequate lesion detection, also in ectopic locations such as the retroesophageal space, within the thyroid, and within the thymus [28, 33, 34, 36, 37].

The aim of our study was to systematically evaluate anatomical MR sequences used in [^18^F]fluorocholine PET/MR for the preoperative localization of parathyroid adenomas in patients with pHPT, and to compare their diagnostic performance. Specifically, our study investigated differences in visualization quality – including lesion conspicuity, margin delineation, and size measurement – between commonly used MR pulse sequences and across various anatomical locations of adenomas. Given the expanding clinical adoption of integrated PET/MR systems, this evaluation is both timely and of clinical relevance [25, 33, 34, 42].

## Materials and methods

### Patients

**Fig. 1:**
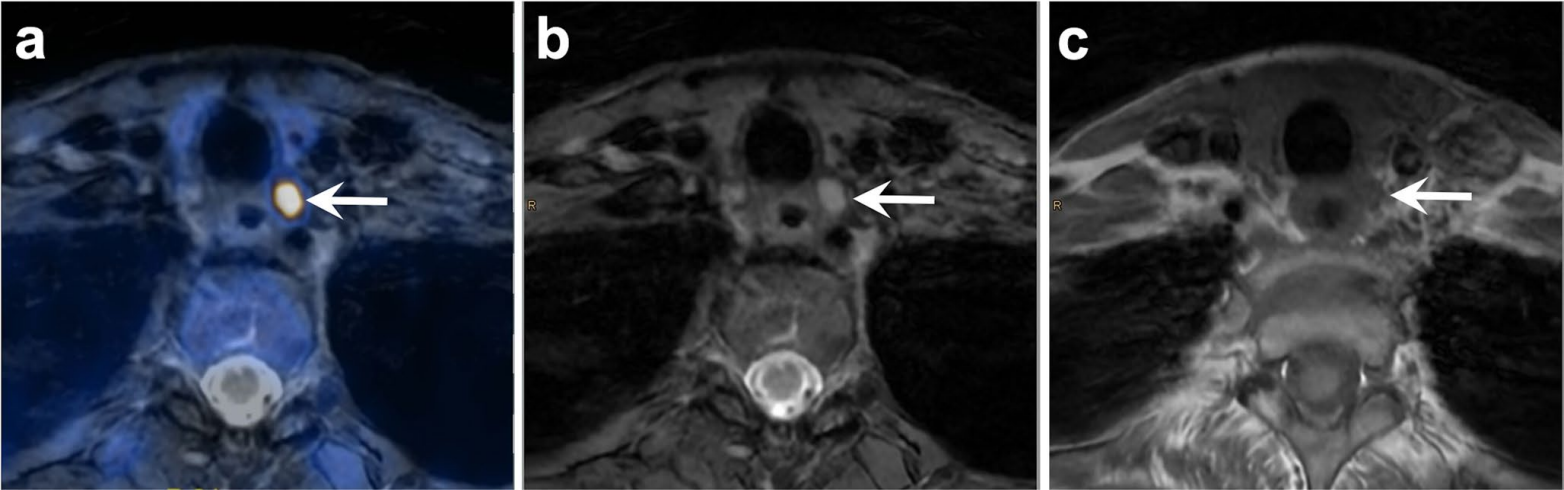
56 year-old woman with pHPT (PTH level 125.1 pg/mL, albumin-corrected serum calcium level 2.65 mmol/L). A (slightly overly) descended left-sided upper pole parathyroid adenoma (arrow; SUV_max_ 19.6, 7.5 x 5.5 x 15.0 mm) is seen on axial fused [^18^F]fluorocholine PET/MR image (**a**), axial T2-weighted flex water MR image (**b**) and axial T1-weighted FSE MR image (**c**)

**Fig. 2:**
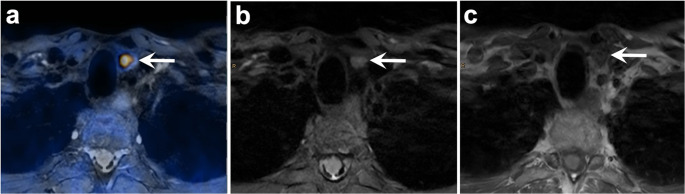
48 year-old man with pHPT (PTH level 123.0 pg/mL, albumin-corrected serum calcium level 2.67 mmol/L). An orthotopic left-sided lower pole parathyroid adenoma (arrow; SUV_max_ 9.4, 8.0 x 5.5 x 11.0 mm) is seen on axial fused [^18^F]fluorocholine PET/MR image (**a**), axial T2-weighted flex water MR image (**b**) and axial T1-weighted FSE MR image (**c**)

The responsible ethics committee approved this retrospective study. The study was conducted in accordance with the Declaration of Helsinki. Only patients who had provided written consent to the use of their medical data for retrospective research purposes were included. Patients were eligible for inclusion if they had a clinically confirmed diagnosis of pHPT and underwent [^18^F]fluorocholine PET/MR imaging at our institution between December 2020 and October 2023. A total of 125 patients met these criteria (Figs. 1 and 2).

**Fig. 3:**
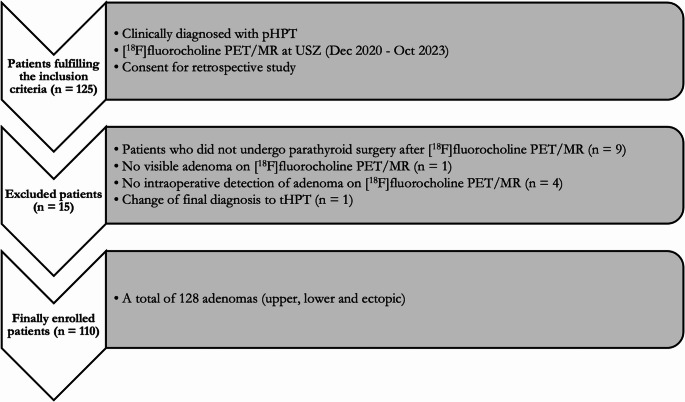
Study population enrolment with inclusion and exclusion criteria

Exclusion criteria were as follows: Patients who did not undergo parathyroid surgery after PET/MR imaging (*n* = 9); cases in which no parathyroid adenoma was identified during surgery (*n* = 4); patients with negative PET/MR findings but with intraoperative detection of an adenoma (*n* = 1); and patients finally diagnosed with tertiary hyperparathyroidism (*n* = 1) (Fig. 3). After applying these criteria, a total of 110 patients were included in the final analysis. Surgical findings identified a total of 128 parathyroid adenomas, of which 118 were orthotopic (84 upper pole and 34 lower pole adenomas (Figs. 1 and 2)), and 10 were truly ectopic.

Since a reliable histopathological distinction between parathyroid adenoma and hyperplasia is not possible, the term “adenoma” is used throughout this manuscript – following institutional convention – to refer to benign, hyperfunctioning parathyroid glands responsible for pHPT [38].

### [^18^F]fluorocholine PET/MR

Hybrid imaging was performed using a SIGNA PET/MR scanner (GE HealthCare, Waukesha, WI) with a magnetic field strength of 3.0 T. Patients received a standardized intravenous dose of [^18^F]fluorocholine (mean activity: 146.9 ± 14.3 MBq), and imaging commenced approximately 50 min after injection [17]. PET data were corrected for random coincidences, scatter, dead time, and attenuation (Table 1).

**Table 1:** Patient demographics

Parameter		All patients (*n* = 110)
Age (years), median (range)	64.5 (20.2–87.4)	
Female sex, *n* (%)	91 (82.7)	
PTH serum level (pg/mL) before PET/MR, median (range), mean ± SD	109.5 (53.2–1391.0), 196.5 ± 290.7	
Adenomas, absolute *n* (upper, lower, ectopic), mean *n* per patient	128 (84, 34, 10), 1.16	

*n*, number; PTH, parathormone; SD, standard deviation

PET images were reconstructed using a 3D time-of-flight ordered subset expectation maximization algorithm (2 iterations and 28 subsets) and block-sequential regularized expectation maximization (beta value of 200). Acquisition and reconstruction parameters included a matrix size of 256 × 256 pixels, an acquisition time of 3 min per bed position, with 2 bed positions per patient, with an axial field of view of 153 mm.

Attenuation correction was performed using an axially acquired T1-weighted (T1w) Dixon-based sequence. This partial-body MR protocol covered the area from the head to the upper abdomen. It included two MR pulse sequences: a T1w liver acquisition with volume acceleration-flexible (LAVA-flex) and a coronal T2-weighted (T2w) single-shot fast spin echo (FSE) sequence.

The regionalized diagnostic MR protocol for the head, neck, and upper mediastinum included the following MR pulse sequences: an axial T1w FSE, coronal T2w with short tau inversion recovery (STIR PROPELLER), sagittal T2w PROPELLER without fat saturation, an axial T2w Iterative Decomposition of water and fat with Echo Asymmetry and Least-squares estimation (IDEAL) with water-only reconstruction (T2w FSE flex water), and an axial T1w LAVA-flex with reconstruction of in-phase and opposed-phase images (Table 2).

**Table 2:** Acquisition parameters of the regionalized MR protocol

Parameter	Axial T2w flex water	Axial T1w FSE	Coronal STIR PROPELLER	Sagittal T2w PROPELLER	Axial T1w LAVA flex
TR/TE (ms)	5120/80	640/7.4	7020/60	6400/124	659/min/full
Flip angle (degree)	111	111	100	142	111
TI	N/A	N/A	200	N/A	N/A
Parallel imaging acceleration factor	2.5	2.5	3	2	2
Slice thickness (mm)	2.5	3.5	3.5	2.5	3
FOV (cm)	20	20	26	22	22
Acquisition matrix (pixels)	356 × 224	300 × 236	280 × 280	340 × 340	280 × 240
Receiver bandwidth (kHz)	83.33	62.5	83.33	62.5	83.33
Fat saturation	IDEAL	N/A	STIR	N/A	IDEAL
Total acquisition time (min)	3:05	2:15	4:54	2:46	2:02

For all sequences using IDEAL methods, images with and without fat suppression are acquired simultaneously. FOV, field of view; IDEAL, iterative decomposition of water and fat with echo asymmetry and least-square estimation; LAVA, liver acquisition with volume acquisition; N/A, not applicable; PROPELLER, periodically rotated overlapping parallel lines with enhanced reconstruction; STIR, short tau inversion recovery; T1w, T1-weighted; T2w, T2-weighted; TI, inversion time

Gadolinium-based contrast agents were not administered, as prior studies have shown no significant diagnostic benefit in this context [39]. More detailed parameters for PET/MR acquisition have been described previously [35, 40, 43].

### Image Analysis

Only the diagnostic MR sequences covering the head, neck, and upper mediastinum were included in the image analysis. These five different MR pulse sequence types were consistently acquired across all patients and available for evaluation in all subjects.

In a preliminary analysis, the diagnostic utility of each MR sequence for detecting parathyroid adenomas was assessed. All adenomas were consistently visualized on both T1w FSE and axial T2w FSE flex water images. In contrast, the coronal T2w STIR failed to depict adenomas in 22 cases and was therefore excluded from further evaluation. Similarly, the sagittal T2w PROPELLER did not provide adequate, clear delineation of adenomas in 27 cases and was therefore excluded. The in-phase reconstruction of the axial T1w LAVA-flex sequence yielded image quality comparable to that of the T1w FSE sequence, but it offered no additional diagnostic value; therefore, it was excluded from further analysis. Based on these findings and a preliminary analysis, the two most suitable MR pulse sequences—axial T1w FSE and axial T2w FSE flex water—were therefore selected for detailed analysis across the entire patient cohort.

All [^18^F]fluorocholine PET/MR exams were independently evaluated by two experienced readers, a radiologists/nuclear medicine physicians (R1: J.A.S.,10 years of experience) and a radiologist (R2: A.G.G.,10 years of experience). For each examination, the presence and location of focal [^18^F]fluorocholine uptake consistent with hyperfunctioning parathyroid tissue were documented. Lesion location was categorized by sidedness (left, right, ectopic).

The MR component of the hybrid PET/MR was used for anatomical correlation and precise localization of PET-positive findings. Each reader independently assessed the MR visibility of every identified adenoma using the following criteria: conspicuity, rated on a 5-point Likert scale (1 = poor conspicuity, barely recognizable; 5 = excellent conspicuity, easy identifiable); delineation, rated on a 5-point Likert scale (1 = poor margin definition; 5 = sharply defined margins); and size (maximum lesion diameter in mm).

To assess interreader agreement, Cohen’s kappa (κ) statistic was calculated. Interpretation of kappa followed the classification proposed by Landis and Koch: κ < 0 = poor agreement, 0.01–0.20 = slight, 0.21–0.40 = fair, 0.41–0.60 = moderate, 0.61–0.80 = substantial, and 0.81–1.00 = almost perfect agreement.

The evaluation of the PET/MR image data was conducted according to current best practice recommendations for the interpretation of [^18^F]fluorocholine PET/MR in pHPT, as outlined in the recent literature [17].

### Statistical Analysis

Statistical analyses were conducted using IBM Statistical Package for the Social Sciences (SPSS) Statistics version 26.0 software (accessed June 2nd, 2025). Continuous variables were reported as mean ± standard deviation (SD) or median (interquartile range, IQR), while categorical variables were reported as frequencies and percentages. To determine whether there was a systematic difference in scoring between the two readers, the Wilcoxon signed-rank test was applied to the ordinal data. Lesion size measurements were also independently assessed by both readers and compared. Additionally, all analyses were repeated in three anatomical subgroups based on adenoma location: (1) orthotopic upper pole adenomas, (2) orthotopic lower pole adenomas, and (3) ectopic adenomas (including retroesophageal, intrathymic, and submandibular locations).

## Results

The patients’ demographic data are summarized in Table 1. For conspicuity, substantial agreement was observed on the T1w FSE sequence (κ = 0.725; *p* < 0.001), while the T2w FSE flex water sequence showed almost perfect agreement (κ = 0.812; *p* < 0.001). Regarding delineation, the T1w FSE sequence demonstrated almost perfect agreement (κ = 0.862; *p* < 0.001), and the T2w FSE flex water sequence showed substantial agreement (κ = 0.777; *p* < 0.001). Based on these results, the interreader scores were averaged to create combined variables for each parameter for subsequent analysis.

No statistically significant differences were observed between the two readers in lesion size measurements for either MR sequence. The Wilcoxon signed-rank test for the T1-weighted FSE sequence yielded a z-value of − 0.070 (*p* = 0.944), indicating no systematic interreader variation. Similarly, for the T2-weighted FSE flex water sequence, the test yielded a z-value of − 0.386 (*p* = 0.700), demonstrating high consistency in size assessment between readers.

Based on the combined reader evaluations, parathyroid adenomas demonstrated significantly higher conspicuity on T2w FSE flex water compared to T1w FSE (mean ± SD: 4.02 ± 1.23 vs. 2.99 ± 1.37, respectively; Wilcoxon signed-rank test, z = −7.227; *p* < 0.001). Similarly, delineation was better on T2w FSE flex water than on T1w FSE (mean ± SD: 3.48 ± 1.26 vs. 2.93 ± 1.45, respectively; Wilcoxon signed-rank test, z = −4.501; *p* < 0.001). Additionally, adenomas measured larger on T2w FSE flex water than on T1w FSE (mean ± SD: 7.25 ± 2.89 mm vs. 6.71 ± 3.02 mm, respectively; Wilcoxon signed-rank test, z = −4.214; *p* < 0.001).

Subgroup analyses stratified by adenoma location revealed the following characteristics: Upper pole adenomas exhibited significantly higher conspicuity on T2w FSE flex water compared to T1w FSE (mean ± SD: 4.22 ± 1.13 vs. 3.13 ± 1.41, respectively; *p* < 0.001), improved delineation (mean ± SD: 3.65 ± 1.23 vs. 3.07 ± 1.49, respectively; *p* < 0.001), and larger measured lesion size (mean ± SD: 7.46 ± 3.17 mm vs. 6.86 ± 3.40 mm, respectively; *p* < 0.001). Similarly, lower pole adenomas demonstrated higher conspicuity on T2w FSE flex water than on T1w FSE (mean ± SD: 3.66 ± 1.39 vs. 2.72 ± 1.27, respectively; *p* < 0.001), better delineation (mean ± SD: 3.32 ± 1.32 vs. 2.868 ± 1.443, respectively; *p* < 0.017), and larger size measurements (mean ± SD: 6.84 ± 2.29 mm vs. 6.30 ± 2.12 mm, respectively; *p* = 0.042).

Ectopic adenomas also demonstrated higher conspicuity on T2w FSE flex water compared to T1w FSE (mean ± SD: 3.65 ± 1.27 vs. 2.80 ± 1.40, respectively; *p* = 0.041). However, there was no significant difference in delineation between T2w FSE flex water and T1w FSE (mean ± SD: 2.65 ± 0.88 vs. 2.65 ± 1.11, respectively; *p* = 1.000), nor in lesion size measurement (mean ± SD: 6.92 ± 2.22 mm vs. 6.92 ± 2.26 mm, respectively; *p* = 0.646).

## Discussion

Our study aimed to evaluate various MR pulse sequences used in clinical [^18^F]fluorocholine PET/MR for localizing parathyroid adenomas in patients with pHPT. Our results demonstrate that the T2w FSE flex water sequence provides significantly superior conspicuity, delineation, and lesion size assessment of adenomas compared to the T1w FSE sequence. In contrast, both sequences identify the same number of adenomas. In the direct sequence comparison, the T2w FSE flex water sequence proved superior to the T1w FSE sequence across all three parameters examined (*p* < 0.001). These differences were statistically significant and also clinically relevant as accurate preoperative detection and precise localization of hyperfunctioning parathyroid tissue are essential for planning minimally invasive parathyroidectomy. These findings support the incorporation of T2w FSE flex water into standardized diagnostic PET/MR protocols for pHPT [44].

In the subgroup analysis, upper pole adenomas exhibited the most favourable imaging characteristics on T2-weighted FSE flex water sequences, highlighting the utility of this sequence, particularly in anatomically less complex regions. Although conspicuity also improved for ectopic lesions using T2-weighted FSE flex water, delineation and size assessment remained identical compared to T1w FSE. This suggests that lesion localization and surrounding anatomical complexity may influence the added diagnostic value of T2-weighted imaging. One plausible explanation is that T2-weighted sequences with fat suppression perform exceptionally well in the upper neck, where fat suppression is more homogeneous and less disturbed by adjacent structures. In this region, parathyroid adenomas appear hyperintense and are thus more easily distinguished from the surrounding tissue. In contrast, suppression becomes increasingly challenging in the lower neck due to adjacent lung parenchyma, osseous structures such as the clavicle and sternum, and pulsation artifacts from large vessels, all of which degrade image quality and limit diagnostic confidence for lower pole adenomas [45, 46].

This result underscores the ongoing diagnostic challenges posed by ectopically located adenomas – especially those in retroesophageal or intrathymic locations – where proximity to vascular and aerated structures, along with motion artifacts, may impair precise border definition.

These findings suggest that while T2w fat-suppressed sequences enhance lesion visibility overall, their diagnostic performance is influenced by anatomical location, with diminished value in complex mediastinal topographies that pose challenges to fat suppression techniques. Accordingly, additional strategies – such as multiparametric imaging or advanced motion-compensated methods – may be necessary to optimize lesion detection and characterization in these challenging regions.

The simultaneous acquisition and combined evaluation of PET/MR data represent a methodological advantage, enhancing metabolic lesion characterization through direct spatial correlation. MR images were independently assessed by two experienced imaging specialists trained and board-certified in radiology. The resulting interreader agreement ranged from substantial to almost perfect (κ = 0.725–0.862), reflecting the high reproducibility and robustness of the diagnostic evaluations. The absence of significant interreader differences in lesion size assessments for both T1w FSE and T2w FSE flex water sequences further supported the reliability of the measurements.

It is particularly noteworthy that the axial T2w FSE flex water sequence was the only MR sequence that consistently demonstrated both sensitivity and reliability in visualizing parathyroid tissue. When combined with the functional information from [^18^F]fluorocholine PET, this results in a hybrid imaging method with high diagnostic value and direct relevance for surgical planning [36, 47].

An additional clinically important aspect is the ability to determine the spatial relationship of the adenomas to the trachea (anterior vs. posterior), which facilitates accurate preoperative localization and designation of adenomas as upper or lower gland pole. This functional topographic information significantly influences the choice and approach of surgical intervention and may be further enhanced by the implementation of standardized hybrid PET/MR imaging.

The results of our study support the routine inclusion of the axial T2w FSE flex water sequence in [^18^F]fluorocholine PET/MR protocols for evaluating pHPT. While several studies have advocated for the use of 4D-MR — dynamic contrast-enhanced MR imaging — for detecting parathyroid adenomas due to its ability to capture characteristic enhancement patterns, our findings suggest that such approaches may not be necessary in the context of PET/MR [22, 23, 39, 46, 48]. We demonstrate that standard anatomical MR sequences without contrast, when interpreted alongside [^18^F]fluorocholine PET, are sufficient for lesion localization and characterization. The diagnostic value in our setting primarily stems from the PET component, which enables the precise identification and functional characterization of parathyroid adenomas. The MR component mainly serves to provide anatomical correlation, especially with regard to lesion location and vascular relationships. While we cannot entirely exclude the possibility that contrast-enhanced MR could further improve lesion characterization in selected cases, our data indicate that it is generally dispensable within an integrated PET/MR workflow [34].

Owing to their intrinsically high T2 signal, parathyroid lesions typically appear hyperintense relative to surrounding tissues on high–b-value diffusion-weighted images (DWI) [49]. However, they generally do not demonstrate true diffusion restriction, and thus are difficult to distinguish from neighboring lymph nodes, which is the main reason why DWI is generally not used in the work-up of hyperparathyroidism. Additionally, since anatomical detail is sparse, DWI is also not useful for the morphological characterization of lesions.

The results of our study may also inform standalone MR imaging, supporting the differentiation of physiological structures such as lymph nodes from parathyroid adenomas through optimized sequence selection.

The integration of artificial intelligence-based tools for automated lesion detection and classification may further enhance the reproducibility and diagnostic performance of PET/MR in pHPT. Deep learning algorithms trained on hybrid imaging data have the potential to assist in the identification of subtle lesions and reduce reader dependency. Although initial developments in this field are promising, their clinical implementation requires access to large, annotated datasets and rigorous external validation [50].

For clinical practice – particularly in Switzerland, where PET/MR is increasingly integrated into routine clinical care – our data offer an essential foundation for standardizing the selection of MR sequences. Future investigations should also incorporate economic considerations. Although access to PET/MR is currently limited primarily to university centres, improved preoperative localization may shorten surgical duration and reduce unnecessary exploratory procedures or repeat surgery, potentially shifting the long-term cost-benefit balance in favour of hybrid PET imaging [51–53].

Nevertheless, further studies are essential to validate these findings. Prospective trials involving external cohorts, as well as comparative analyses with PET/CT, could help establish broader diagnostic validity and generalizability. In particular, the assessment of ectopic adenomas remains a diagnostic challenge, and further research should focus on overcoming current limitations in morphological differentiation from other physiologic or pathologic structures encountered in these anatomical areas.

Despite the promising results, several limitations of our study must be acknowledged. First, the retrospective design may introduce inherent selection bias and limit the degree of standardization in imaging parameters and acquisition protocols. However, the exams analysed followed a fixed standardized imaging protocol by default. Second, although the overall sample size was relatively large for a single-centre study, the number of truly ectopic adenomas was small, thereby restricting statistical power for subgroup analyses in this cohort. Additionally, variability in lesion-to-background contrast and susceptibility to fat suppression failure and motion artifacts – particularly in lower neck and mediastinal locations – may have negatively impacted lesion assessment in these anatomically complex regions, contributing to the lower performance observed in this group [46]. Third, only a limited subset of MR pulse sequences from the standard clinical protocol was included in the final analysis, and these had a slightly different slice thickness. Other sequences were excluded during preliminary evaluation, and no advanced or research-specific MR sequences were employed. In addition, the increasing incorporation of quantitative parameters, such as maximum standardized uptake value (SUV_max_) from the PET component or signal intensity ratios (e.g., lesion-to-muscle ratio) from the MR component, may contribute to a more objective assessment of features like conspicuity, although there is currently only limited data to support semiquantitative SUV_max_ analysis in parathyroid [^18^F]fluorocholine PET [54]. This is particularly relevant given the inter-individual variability inherent in image interpretation and the limitations of purely visual assessment, especially in cases with borderline findings or multiglandular disease. Lesions in our cohort were classified as “adenomas,” a term that also encompasses hyperplasia, as reliable differentiation between these entities is not possible on either histopathology or imaging. This approach is considered appropriate, given that both conditions produce largely identical clinical manifestations. Finally, no proper standard of reference for adenoma size was available since adenomas are characterized by the weight of surgical specimens, which typically contain surrounding adipose tissue. While these limitations must be acknowledged, this study provides the most comprehensive evaluation of MR sequence optimization in parathyroid PET/MR imaging. These findings offer immediate clinical applicability and establish a basis for further technological advancements.

## Conclusion

In conclusion, our study demonstrates that the axial T2w FSE flex water sequence provides robust diagnostic performance for detecting parathyroid adenomas across different evaluation parameters, with particularly notable advantages in imaging orthotopic lesions. These findings support its establishment as a standard diagnostic sequence within hybrid [^18^F]fluorocholine PET/MR protocols and may also inform standalone MR imaging.

## Data Availability

The datasets generated during and/or analysed during the current study are available from the corresponding author on reasonable request.

## Abbreviations

CT: Computed tomography
FSE: Fast spin echo
IDEAL: Iterative decomposition of water and fat with echo asymmetry and least-squares estimation
LAVA-flex: Liver acquisition with volume acceleration-flexible
MBq: Megabecquerel
MR: Magnetic resonance imaging
PET: Positron Emission Tomography
PROPELLER: Periodically rotated overlapping parallel lines with enhanced reconstruction
pHPT: Primary hyperparathyroidism
SPECT: Single-photon emission computed tomography
STIR: Short tau inversion recovery
SUV_max_: Maximum standardized uptake value
